# 605. Bridging Burn Care: A Pharmacists Role in Inpatient to Outpatient Transitions of Care

**DOI:** 10.1093/jbcr/irag033.416

**Published:** 2026-04-06

**Authors:** Janie Faris, Holly Coco, Spencer Simko, Rebecca Coffey

**Affiliations:** Parkland Health, Grapevine, TX; University of Texas Southwestern Medical Center, Dallas, TX; University of Texas Southwestern Medical Center, Dallas, TX; Parkland Health, Dallas, TX

## Abstract

**Introduction:**

In 2023, the pharmacy department at a regional burn center launched a novel Transition of Care (TOC) initiative to improve the continuity of care for burn patients. Pharmacist-led TOC models are well-established in chronic disease management, with limited application in burn care. The objective of this study was to evaluate the impact of pharmacist interventions on TOC outcomes for burn patients, with a focus on medication safety and access.

**Methods:**

A retrospective cohort study compared burn patients pre- (Oct 2022–Oct 2023) and post-TOC implementation (Nov 2023–Nov 2024). Key interventions included: (1) a dedicated TOC pharmacist for discharge and outpatient follow-up; (2) multilingual medication education on pain, sleep, pruritus, bowel regimens, and pediatric dosing; (3) structured medication reconciliation at admission/discharge; (4) post-discharge pharmacy follow up calls; (5) a collaborative practice agreement with the burn surgery group; and (6) expanded medication assistance programs, including free discharge medications for eligible patients (acetaminophen, ibuprofen, senna, docusate, polyethylene glycol, glycerin suppositories).

**Results:**

A total of 482 patients were included in the pre-implementation group and 842 in the post-implementation group. The pharmacy team developed medication educational materials in five languages that can be included in the discharge instructions. Nearly 50% of patients were missed for follow-up calls due to challenges identifying burn patients and pharmacy staffing. Readmission rates remained stable, but over half of patients were uninsured or underinsured, making free discharge meds impactful. The collaborative agreement streamlined medication refills and referral processes for chronic illnesses, improving continuity of care. A summary of pharmacist interventions is presented in Table 1.

**Conclusions:**

Pharmacy-led TOC model in burn care has demonstrated meaningful improvements in patient care and revealed opportunities for growth. Future directions include development of real-time reports to identify all burn patients, expanding formulary medications free discharge, increasing TOC pharmacy staffing, and earlier engagement with inpatient medication assistance programs.

**Applicability of Research to Practice:**

readmission rates post burn pt discharge occurs frequently. There is limited data available on implementing a transition of care practice with pharmacy (from inpatient to outpatient) in burn patients. This abstract provides benefits observed in starting a new program and areas of where additional improvement is available.

**Funding for the study:**

N/A.

